# Identifying risk factors and detection strategies for adolescent depression in diverse global settings: A Delphi consensus study

**DOI:** 10.1016/j.jad.2020.09.098

**Published:** 2021-01-15

**Authors:** Syed Shabab Wahid, Katherine Ottman, Raya Hudhud, Kamal Gautam, Helen L. Fisher, Christian Kieling, Valeria Mondelli, Brandon A. Kohrt

**Affiliations:** a Division of Global Mental Health, George Washington University, Washington DC, United States; b Department of Environmental and Occupational Health, George Washington University, Washington DC, United States; c Transcultural Psychosocial Organization Nepal (TPO Nepal), Baluwatar, Kathmandu, Nepal; d Social, Genetic & Developmental Psychiatry Centre, Institute of Psychiatry, Psychology & Neuroscience, and ESRC Centre for Society and Mental Health, King's College London, London, United Kingdom; e Department of Psychiatry, Universidade Federal do Rio Grande do Sul; and Child & Adolescent Psychiatry Division, Hospital de Clínicas de Porto Alegre, Porto Alegre, RS, Brazil; f Department of Psychological Medicine, Institute of Psychiatry, Psychology & Neuroscience, King's College London, London, United Kingdom

**Keywords:** adolescent, depression, Delphi, risk, detection, prevention

## Abstract

**Background:**

Adolescence represents a vulnerable period for the onset of depression. Globally, there is a need to better understand risk factors for adolescent depression to inform policies for effective prevention initiatives.

**Methods:**

A Delphi consensus study was conducted on risk factors, early signs, and detection strategies for adolescent depression in global settings. Over 3 survey rounds, global experts formulated and ranked these variables for (1) specificity for adolescent depression and (2) feasibility of measurement (round 1, n=21 participants; rounds 2 and 3, n=17). We calculated Smith's salience index as a measure of consensus. Interviews were conducted with 10 participants to elicit qualitative reflections on the ranking results, and on the influence of cultural and contextual factors on depression risks.

**Results:**

Thirty-one risk factors for adolescent depression were generated. Panelists ranked three as highly specific and highly feasible to measure: family history of depression, exposure to bullying, and a negative family environment. Six were ranked as modestly specific and highly feasible: physical illness or disability, female sex, bereavement, trauma exposure, substance abuse, and low self-esteem. An additional 5 items were modestly specific and modestly feasible: social difficulties, academic stress, poverty, loss of family, and cognitive distortions. Five symptoms were at least modestly specific and feasible to measure: mood changes, loss of interest, social isolation, suicidality, and sleep changes. Schools were considered the most feasible place for screening.

**Limitations:**

The participants were not representative of all countries and cultural regions.

**Conclusions:**

This study offers a profile of risk factors developed and prioritized by experts to inform a research agenda for risk, identification and prevention of adolescent depression across global settings.

## Highlights

• Global experts ranked biopsychosocial risk factors for adolescent depression
• Risk factors include female sex, family history, physical illness, bullying
• Mood changes and loss of interest are measurable early signs of adolescent depression
• Culture influences the types and specificity of adolescent depression risk factors

## 1 INTRODUCTION

Globally, depression is one of the leading causes of illness and disability among adolescents (Belfer, 2008). Although half of all mental health conditions start by 14 years of age, most cases remain undetected and untreated during adolescence and into early adulthood (Thapar, Collishaw, Pine, & Thapar, 2012). Not addressing mental health conditions during adolescence impacts future physical and mental health and limits opportunities to lead fulfilling lives as adults (Patton et al., 2016; Thapar et al., 2012). With depression comprising the greatest burden of all mental health conditions worldwide, treatment alone is insufficient to address this problem on a global scale (Andrews, Issakidis, Sanderson, Corry, & Lapsley, 2004; Friedrich, 2017; Whiteford et al., 2013). Therefore, increasing identification of depression early in adolescence and implementing preventive strategies should be highly prioritized for addressing this global burden (Kieling et al., 2019).

Understanding the biopsychosocial risk factors that can predict the onset of depression, and the protective factors which can inform measures for preventing its manifestation and severity, are important steps towards achieving this goal. Recent research has shifted towards efforts addressing early detection and prevention, as several reviews have gathered promising results of potentially curbing the occurrence or early onset of depression in adolescence (Cairns, Yap, Pilkington, & Jorm, 2014; Cairns, Yap, Reavley, & Jorm, 2015; van Zoonen et al., 2014; Werner-Seidler, Perry, Calear, Newby, & Christensen, 2017; Yap et al., 2016). However, most prevention or early detection studies, current systematic reviews, and longitudinal studies on adolescent risk factors are primarily conducted in westernized, educated, industrialized, rich and democratic (WEIRD) societies, with a large gap in the literature for low- to middle-income countries (LMIC) (Cairns et al., 2014; Dunn & Goodyer, 2006; Kim-Cohen et al., 2003; Stirling, Toumbourou, & Rowland, 2015; Yap, Pilkington, Ryan, Kelly, & Jorm, 2014).

Recently, cohorts from LMICs have been used to evaluate the predictive power of constellations of risk factors among adolescents for future development of depression (Botter-Maio Rocha et al., 2020; Brathwaite et al., 2020). However, the constellation of risk factors studied have been primarily informed by assumptions from WEIRD populations (Cairns et al., 2014; Kieling et al., 2019; Kieling et al., 2011; Pedersen et al., 2019). This is problematic for three reasons: first, risk factors may be interpreted differently across cultural context; second, risk factors may have different impacts across populations; and third, the feasibility of measuring risk factors varies across settings. Therefore, efforts are needed to develop constellations of risk factors that are informed by diverse global perspectives with particular attention to LMIC, where more than 90 percent of the world's youth reside (Belfer, 2008; Erskine et al., 2015; Kieling et al., 2011; Leckman & Leventhal, 2008).

To inform a more globally representative agenda on studying risk factors among adolescents worldwide, we conducted a Delphi panel consensus study to solicit opinions from global experts in the field of adolescent depression. We collected perspectives on biological, psychological, and environmental risk factors as well as early signs of adolescent depression and detection strategies.

## 2 METHODS

### 2.1 Objectives

This study is part of a larger integrated research portfolio being implemented by the Identifying Depression Early in Adolescence (IDEA) research consortium comprised of psychiatrists, neuroscientists, epidemiologists and anthropologists from Brazil, Nepal, Nigeria, the UK, and United States, conducting multi-disciplinary research on adolescent depression risk and prevention (Kieling et al., 2019). This Delphi study is one aspect of a multi-component qualitative study implemented under the IDEA project. The IDEA Delphi objectives are presented in Table 1. Methods for all qualitative components of the IDEA project and the *a priori* Delphi design for this study are available in a published protocol (Wahid et al., 2020).

**Table 1:** Objectives of IDEA qualitative study and role of Delphi study

Objectives of multi-component IDEA qualitative study	Role of Delphi activity with international experts
Objective 1: To investigate cultural concepts of depression, depression risk, and health systems	Experts commented on risk factors in different cultural settings
Objective 2: To establish the feasibility, acceptability, and perceived utility of a risk calculator for adolescent depression	Experts commented on feasibility of risk assessment and early detection methods
Objective 3: To establish the feasibility and acceptability of biological psychiatry research in low- and middle-income countries	Experts commented on specificity and feasibility of risk factors in different cultural contexts

### 2.2 Delphi method

The Delphi method is a consensus building approach that allows for the systematic generation and scoring of research priorities using predetermined criteria, and has been widely used in depression research (Cairns et al., 2015; Grunder et al., 2017; Jones & Hunter, 1995; Langlands, Jorm, Kelly, & Kitchener, 2008; Morgan & Jorm, 2009; Yap et al., 2014). The method entails a process of collecting expert opinion through multiple survey panels, with the opportunity of providing facilitated feedback to respondents preceding each survey round, to lead to consensus on the topic of inquiry. The Delphi method is especially useful in guiding a developing field of research where gaps in literature exist, by providing a ‘state of the field’ expert recommendation as a foundation for further action (Fish & Busby, 1996; Keeney, Hasson, & McKenna, 2011).

Recently, modified approaches to the Delphi method have been utilized to reduce panel attrition, through the use of fewer survey rounds, and the incorporation of quantitative and qualitative methods (Freitas, Williams-Reade, Distelberg, Fox, & Lister, 2016; Keeney et al., 2011). In this study, the Delphi method was used to address biopsychosocial risk factors, early signs of adolescent depression, and strategies for detection. Global experts were engaged using mixed methods over three survey rounds.

### 2.3 Recruitment and panel formation

Recruitment for the study was done by sending out invitations to an initial pool of 100 global experts in adolescent depression. Experts were identified through a literature scan of highly cited authors from peer-reviewed academic journals focused on adolescent mental health. The list was supplemented through recommendations from the professional networks of the IDEA research consortium members, with specific efforts to recruit LMIC experts. The recruitment rationale for Delphi studies follows a purposive approach of soliciting and recruiting those individuals who hold expertise on the subject matter under investigation and does not necessitate a representative sample. Standard panel formation guidelines for consensus studies indicate insignificant gains in reliability with more than 15 panelists, and a decrease in reliability with fewer than six panelists (Murphy et al., 1998). Accordingly, our recruitment efforts aimed to establish a panel of 15 or more participants. Ethical approval for the IDEA Delphi study was provided by the institutional review board of the George Washington University. All data collection procedures were conducted with informed consent and voluntary participation of respondents.

### 2.4 Questionnaire development and administration

The development of the study instruments was informed by a biopsychosocial approach to depression, and by calls for research that contextualizes experiences of distress and related social determinants across global settings (Engel, 1977; Kirmayer & Pedersen, 2014). The questionnaires for the three survey rounds were administered in early, middle, and late 2019.

The Round-1 open-ended questionnaire (Supplementary appendix A) was active for 30 days, and panelists were sent one email reminder to complete it. The primary focus of the survey was for panelists to describe salient biological, psychological, and environmental risk factors for depression among adolescents. In addition, panelists provided responses on early signs of depression and early detection strategies. The survey also contained panelist-specific demographic questions including their experience and range of expertise.

In Round-2, panelists were asked to rank finalized items (Supplementary appendix B) from Round-1 (biological, psychological, and environmental risk factors, early signs and detection strategies), according to two dimensions: (1) feasibility of measurement; and (2) specificity to adolescent depression. Panelists were asked to rank the items in consideration to the context they work in, in descending order, to indicate ranking hierarchy. Respondents were asked to rank items based on their theoretical knowledge, empirical research evidence, clinical experience of depression treatment, and any personal experiences. Operational definitions from the literature for each item were available to the panelists during the survey. A final open-ended question asked panelists to comment on how cultural and contextual elements may influence their ranking. Round-2 was active for 30 days and respondents were sent one email reminder after two weeks.

In Round-3, summary results from Round-2 were shared with respondents. These summary tables indicated items that had reached consensus and were endorsed by the panel, and those that did not. All panelists were provided with the opportunity to reconsider their rankings at this stage.

After the 3 rounds, we adopted a methodological innovation for Delphi studies, and introduced qualitative key-informant interviews to capture nuanced understanding of the panel rankings. The use of qualitative interviews in a Delphi study is fairly uncommon in the literature thus far, and has been primarily used to clarify discrepancies and add meaning to the data collected (Blow & Sprenkle, 2001; Sori & Sprenkle, 2004). All panelists who completed Round-2 were invited to participate in the qualitative interviews. Interviews lasted approximately 30 minutes and were audio recorded (Supplementary appendix C: qualitative interview guide).

The open-ended survey and ranking exercise were implemented via Qualtrics, a web survey software (Qualtrics, 2019). Rounds 1-3 communication with panelists was carried out over email. Key-informant interviews were conducted via web-conferencing. Panelists received information on the Delphi methodology for their reference at the initiation of the surveys.

### 2.5 Data analysis

#### 2.5.1 Round-1-Open ended survey

The open-ended responses from Round-1 yielded data in the form of narratives and lists of the biological, psychological, and environmental risk factors; early signs; and detection strategies for adolescent depression. Using a data reduction approach informed by empirically grounded construction of typologies, this raw information was analyzed to assimilate and collate similar items (Kluge, 2000). Respondent-driven variable lists were supplemented with other salient variables from preliminary results of a systematic literature review of biopsychosocial risk factors of adolescent depression, a complementary component of IDEA research (Pedersen et al., 2019). These variables comprised the final lists for the ranking exercise of Round-2.

#### 2.5.2 Round-2 Ranking exercise

After the conclusion of Round-2, the ranked lists for each category were analyzed statistically to determine salience as an indicator of panel consensus. The data was entered into FLARES software for cultural analysis (Wencelius, Garine, & Raimond, 2017). We generated a salience index (Smith's *S*), which is a function of the frequency of an item, and its average rank, across the total number of lists. Smith's S index is defined as: *S* = ((*L*- *R_x_* + 1)/*L*)/*N*, where *L* is the length of each list, *R_x_* is the rank of item *X* in the list, and *N* is the number of lists in the sample (Smith & Borgatti, 1997). The Smith's S index generates values between 0 and 1, with values closer to 1 indicating higher consensus. We ran frequency analyses of the Smith's salience indices to visualize the distribution. We selected a cut-off of 0.5 and above, representing the top 20 percent of salience values in the sample, as ‘highly salient.’ We selected a cut-off of below 0.30, which represents the bottom 50 percent of values, as ‘low salience’. For the 30 percent of values between 0.30 and 0.49, we referred to this as ‘moderate salience.’

#### 2.5.3 Round-3 Re-ranking exercise

For Round-3 we compiled summary results tables (mean, median, Smith's *S*) from Round-2 and shared these with the panelists by email. Panelists were requested to review the preliminary results and had the option to revise their rankings. One email reminder was sent two weeks after the results were shared. None of the panelists opted to change their ranking.

#### 2.5.4 Qualitative interviews

Using a mix of deductive and inductive approaches, we developed key-informant interview guides. Based on ranking exercise results, we formulated questions about panelists’ rationale for responses, deviations of individual responses from panel averages, and clarifications for discrepancies. Due to cultural heterogeneity in global settings, we included questions probing the influence of culture on risk factors, early signs, and detection strategies. We used thematic analysis to analyze data following an *a priori* deductive codebook approach. Six steps to thematic analysis were implemented (Braun & Clarke, 2006). The codebook was composed of the Delphi ranking categories and specific items within the categories identified in the quantitative analysis. We directly charted data from the transcripts into results matrices organized according to the codebook. For certain domains, thematic saliency was a function of endorsement across the sample, while for other relevant domains, it was determined by the unique context-specific insights of particular panelists (Malterud, Siersma, & Guassora, 2016).

## 3 RESULTS

In the following section we present results from the Delphi surveys and qualitative interviews. Salience indices are reported from the results of Round-2, as no respondents opted to alter their rankings in Round-3.

### 3.1 Panelist demographics & ranking survey items

The demographics and expertise of panelists are presented in Table 2. Respondents (n=21) from Australia, Brazil, Chile, Croatia, Nepal, Singapore, Sri Lanka, Trinidad and Tobago, Uganda, and the United States participated in the first round of the survey. Two-thirds had 10 or more years of experience in adolescent mental health, 58 percent were from LMIC, 48 percent were female, and 25 percent had expertise in LGBTQ populations.

**Table 2:** Panelist demographics and expertise for Round-1 (n=21)

Demographics/Expertise	Panelists	
	n	%
**Region**		
** **South Asia	7	33
Latin America and the Caribbean	5	29
** ** East Asia and the Pacific	4	19
** ** Sub-Saharan Africa	2	10
** ** North America	2	10
** **Europe and Central Asia	1	5
**Gender**		
** ** Female	10	48
** **Male	11	52
**Experience**		
** **<10 Years	7	33
** **10-20 Years	10	48
** **>20 Years	4	19
Expertise – Disorders*		
** **Mood disorders	15	75
Anxiety disorders	10	50
** **Trauma-related disorders	3	15
Developmental disorders	2	10
** **Neurological disorders	5	25
** ** Psychotic disorders	1	5
Expertise – Population*		
** **Children	16	80
Adolescents	18	90
LGBTQ populations	5	25
Refugee populations	4	20
**Expertise – Geographical focus**		
** **High-income countries	9	42
Low- and middle-income countries	12	58

⁎ Respondents could select multiple options

The biological risk factors identified in Round-1 were *endocrine factors; family history; female sex; gender dysphoria; inflammation; malnutrition; physical illness, injury or disability*; and *substance abuse*. Psychological risk factors included *cognitive distortions; emotional abuse; emotional reactivity; exposure to trauma; ineffective coping; loss and bereavement; low self-esteem; neglect and deprivation; other mental disorders; physical abuse*; and *sexual abuse*. Environmental risk factors included *academic stressors; bullying; decreased access to resources; discrimination; environmental toxins; family environment; gender roles and expectations; humanitarian emergencies; loss of family; migration issues; poverty*; and *social relationship issues*. Of these variables, *inflammation* and *suicidality* were supplemented from the literature review. Additionally, respondents had raised ‘*abuse*’ as a single category – we disaggregated abuse into ‘*sexual abuse*,’ ‘*emotional abuse*,’ and ‘*physical abuse*.’ (Of note, respondents offered these risks according to biological, psychological, and environmental domain headings. We did not assign specific risks to categories, and we are aware that some category placements, e.g., gender dysphoria as a biological risk factor, are controversial across different cultures and context.)

Early signs of adolescent depression included *academic decline; changes in appetite; changes in energy; changes in mood; changes in sleeping patterns; cognitive difficulties; loss of interest; social isolation; somatic complaints; substance abuse*; and *suicidality and/or self-harm*. Finally, early detection strategies included: *education and awareness; health facility detection; home-based detection*; and *passive monitoring technology; screening tests*; and *school-based detection*.

We conducted comparative analyses of means and medians between high-income countries and LMIC panelist participants and did not detect any significant differences or trends in the results.

### 3.2 Ranking exercise and qualitative interviews

#### 3.2.1 Biological risk factors

**Figure 1:**
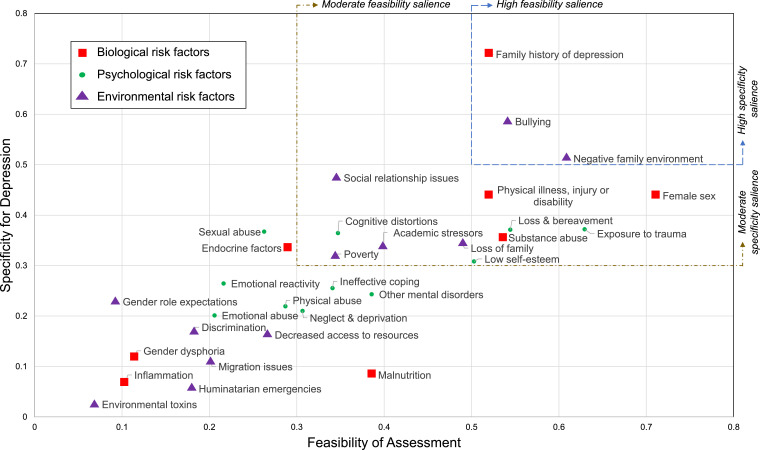
Biological, psychological, and environmental risk factors plotted by Smith's Salience Indices for specificity for adolescent depression and feasibility of assessment. Y-axis: higher scores represent greater ranking of specificity for adolescent depression; X-axis: higher scores represent greater ranking for ease of feasibility to assess the risk factor in the respondents’ context.

When addressing biological risk factors, the panel endorsed 4 items as highly salient for feasibility of measurement, and 1 item as moderately salient (See Table 3 and Figure 1). ‘Female sex’ was ranked the highest (S = 0.7103) followed by ‘substance abuse’ (S = 0.5338), ‘physical illness, injury or disability’ (S = 0.5211) and ‘family history’ (S = 0.5186). ‘Family history’ was the sole item ranked as highly salient for specificity to adolescent depression (S = 0.7206), while 4 items (female sex; physical illness, injury and disability; substance abuse; endocrine factors) achieved moderate consensus among the panelists. The top 4 items for both feasibility and specificity were identical, although ranking order and salience varied slightly.

**Table 3:** Feasibility and specificity of biological, psychological and environmental risk factors of adolescent depression

Feasibility of Measurement				Specificity to Adolescent Depression			
Item	Smith's S Index	Frequency (%)	Average Rank	Item	Smith's S Index	Frequency (%)	Average Rank
***Biological Risk Factors***							
Female sex⁎⁎	0.7103	76.5	1.390	Family history⁎⁎	0.7206	82.4	1.429
Substance abuse⁎⁎	0.5338	76.5	2.850	Female sex*	0.4387	70.6	2.667
Physical illness, injury or disability⁎⁎	0.5211	88.2	3.270	Physical illness, injury or disability*	0.4387	70.6	2.583
Family history⁎⁎	0.5186	76.5	2.920	Substance abuse*	0.3554	64.7	3.182
Malnutrition*	0.3848	76.5	4.000	Endocrine factors*	0.3333	64.7	3.364
Endocrine factors	0.2897	64.7	4.360	Gender dysphoria	0.1225	23.5	4.500
Gender dysphoria	0.1152	41.2	6.290	Malnutrition	0.0833	23.5	5.500
Inflammation	0.1029	35.3	6.670	Inflammation	0.0662	11.8	4.500
***Psychological Risk Factors***							
Exposure to trauma⁎⁎	0.6295	88.2	3.267	Exposure to trauma*	0.3720	58.8	3.200
Loss & bereavement⁎⁎	0.5444	64.7	2.091	Loss & bereavement*	0.3711	58.8	3.200
Low self-esteem⁎⁎	0.5029	82.4	4.143	Sexual abuse*	0.3672	52.9	3.222
Other mental disorders*	0.3858	58.8	3.900	Cognitive distortions*	0.3645	58.8	3.700
Cognitive distortions*	0.3472	64.7	5.636	Self-esteem*	0.3082	47.1	3.125
Ineffective coping*	0.3410	64.7	5.273	Emotional reactivity	0.2645	47.1	4.250
Neglect & deprivation*	0.3066	64.7	5.455	Ineffective coping	0.2553	35.3	3.000
Physical abuse	0.2870	58.8	5.500	Other mental disorders	0.2430	47.1	5.250
Sexual abuse	0.2627	58.8	6.100	Physical abuse	0.2193	41.2	4.571
Emotional reactivity	0.2163	52.9	6.778	Neglect & deprivation	0.2101	47.1	5.000
Emotional abuse	0.2059	47.1	6.875	Emotional abuse	0.2012	41.2	4.857
***Environmental Risk Factors***							
Family environment⁎⁎	0.6084	94.1	3.438	Bullying⁎⁎	0.5858	82.4	3.000
Bullying⁎⁎	0.5415	82.4	3.929	Family environment⁎⁎	0.5123	70.6	2.667
Loss of family*	0.4898	64.7	3.000	Social relationship issues*	0.4740	64.7	2.636
Academic stressors*	0.3988	58.8	3.300	Loss of family*	0.3432	52.9	3.111
Social relationship* issues	0.3454	76.5	5.000	Academic stressors*	0.3399	64.7	4.273
Poverty*	0.3445	47.1	3.500	Poverty*	0.3196	35.3	1.500
Decreased access resources	0.2665	47.1	4.875	Gender role expectations	0.2286	52.9	4.667
Migration issues	0.2021	41.2	5.286	Discrimination	0.1677	41.2	5.714
Discrimination	0.1829	58.8	7.200	Decreased access resources	0.1628	35.3	5.833
Humanitarian emergencies	0.1796	29.4	5.000	Migration issues	0.1085	29.4	7.200
Gender role expectations	0.0926	29.4	8.400	Humanitarian emergencies	0.0561	23.5	8.750
Environmental toxins	0.0686	17.6	8.333	Environmental toxins	0.0250	11.8	10.00

⁎⁎ indicates high consensus

⁎ indicates moderate consensus

**Table 4:** Feasibility and specificity of early signs of adolescent depression

Feasibility of Measurement				Specificity to Adolescent Depression			
Item	Smith's S Index	Frequency (%)	Average Rank	Item	Smith's S Index	Frequency (%)	Average Rank
Academic decline⁎⁎	0.5983	88.2	3.600	Changes in mood⁎⁎	0.5632	70.6	2.167
Somatic complaints*	0.4528	64.7	3.818	Loss of interest*	0.4553	58.8	2.500
Changes in mood*	0.4471	58.8	3.200	Changes in energy*	0.4269	58.8	2.800
Changes in sleeping patterns*	0.4412	76.5	4.538	Social isolation*	0.4266	64.7	3.727
Loss of interest*	0.4283	70.6	4.583	Suicidality and/or self-harm*	0.3853	64.7	4.182
Suicidality and/or self-harm*	0.3918	82.4	5.000	Changes in sleeping patterns*	0.3573	76.5	4.231
Social isolation*	0.3727	76.5	5.769	Academic decline	0.2769	58.8	5.300
Changes in energy	0.2878	52.9	5.556	Somatic complaints	0.2471	47.1	5.125
Changes in appetite	0.2870	47.1	4.875	Cognitive difficulties	0.1930	41.2	6.000
Cognitive difficulties	0.2692	58.8	6.800	Changes in appetite	0.1599	41.2	6.143
Substance abuse	0.2591	70.6	7.000	Substance abuse	0.0672	29.4	8.800

⁎⁎ indicates high consensus

⁎ indicates moderate consensus

**Table 5:** Feasibility of early detection strategies of adolescent depression

Feasibility of Measurement			
Item	Smith's S	Frequency (%)	Average Rank
School based⁎⁎	0.6469	93.8	2.333
Screening tests⁎⁎	0.5365	81.2	2.538
Education awareness⁎⁎	0.5198	81.2	2.462
Health facility⁎⁎	0.5073	81.2	2.769
Home based*	0.3000	62.5	3.700
Passive monitoring technology	0.1458	31.2	4.000

⁎⁎ indicates high consensus

⁎ indicates moderate consensus

Although ‘family history’ was indicated by the panel as the highest endorsed biological risk factor for specificity to adolescent depression, it was ranked fourth for measurement feasibility. When asked during the qualitative interviews to comment on this, respondents indicated that gaps in knowledge of family history of mental illness persist among adolescents and parents, especially in LMIC, as many family members with a condition may never have been formally diagnosed. Additionally, in many cultures, respondents noted, depression symptoms were not recognized as a medical problem in recent generations. This complicates family history recall:

“We've never been quite sure on how to measure it. One approach is to ask parents of their own personal history (of mental illness) and that of other family members. Reality is that individuals aren't particularly good at recalling when they have had an episode, and studies that have looked at lifetime recall, at the age of 29, comparing DSM diagnoses of depression and anxiety, and only about half of all episodes were recalled. The problem of recall is very difficult.”

One respondent suggested incorporation of local terminology of distress in family history interviews as a possible approach to mitigating this issue. Respondents also stated that stigma silences open disclosure and discussion of mental illness in families:

“Presentation of family history is difficult. Stigma plays a role ... Parents might not say what's going on in the family. They may portray the family situation in better terms, especially in terms of how parents may or may not be supporting children. There may also be abuse, which isn't shared. [It is] difficult to measure what sort of family situation is going on.”

‘Endocrine factors’ emerged as a salient risk factor for adolescent depression specificity. However, it did not get endorsed for measurement feasibility. For girls, puberty is measured via self-report on first menstruation. Respondents stated that for boys, biological changes are rarely documented in practice, and therefore are poorly understood. Comprehensive documentation, such as Tanner Stages, was lacking.

Panel results from the ranking exercise reflected low salience for the connection between inflammation and depression, for both specificity and feasibility. Respondents indicated that while the research investigating depression and inflammation was growing, more robust evidence was needed to establish the link. Better-quality studies, especially employing longitudinal study designs, were necessary:

“I think the issue with the research coming out is that defining the pathway from an enhanced inflammatory response to depression is still very exploratory and the link is not as clear yet, as it could be, or as it might become over time. Even with enhanced inflammation, it's certainly not the only, or perhaps not the central thing that might be happening. It has to co-exist with some other risk factor(s). …I'm not sure clinicians are convinced of how inflammation affects depression, and how significant it is as a contributory factor.”

#### 3.2.2 Psychological risk factors

The ranking of psychological risk factors with regard to specificity for adolescent depression indicated heterogeneity of panel opinions, as no item was endorsed with high salience (Table-**3**). ‘Exposure to trauma’ (*S*=0.372), ‘Loss and bereavement’ (*S*=0.3711), ‘Sexual abuse’ (*S*=0.3672), ‘Cognitive distortions’ (*S*=0.3645), and ‘Self-esteem’ (*S*=0.3082) comprised the top 5 items for specificity, with all receiving moderate endorsement, and having minimal variance in the Smith's index value. ‘Sexual abuse’ and ‘Cognitive distortions’ were the only endorsed items specific to adolescent depression that were not considered feasible to measure.

We queried panelists during interviews on this lack of clear consensus for psychological risk factors for specificity to adolescent depression. Respondents cited that this could be due to several reasons: (1) the evidence on psychological risk factors is not fully established and is derived from mostly cross-sectional studies. As such, more longitudinal studies were needed to generate more robust evidence. Studies from diverse non-WEIRD settings are also necessary to inform a global perspective on depression risk in adolescence. (2) The list of psychological risk factors was reported to be ambiguous and overlapping. Respondents mentioned that there is lack of clarity on whether risk factors were independent or part of a causal pathway. (3) Respondents indicated conceptual uncertainty on whether the items are actually risk factors, or if these items were early manifestations of depression. Finally, (4) the variance of experience, geographical focus, and context in which panelists work was mentioned by interview respondents as possible reasons for the response variance as well:

“Psychological risk factors are not well understood – how these develop. A lot of these risk factors co-exist, and it is difficult to determine which one is more critical, or more central. And the capacity to separate them as a result is also quite difficult...This is too broad a [category] – it contains a lot – lot of things are subsumed in this [category].”

#### 3.2.3 Environmental risk factors

For environmental risk factors, 6 items were endorsed for specificity to adolescent depression. ‘Bullying’ (*S*=0.5858), and ‘Family environment’ (*S*=0.5123) were endorsed as highly salient, while ‘Social relationship issues’ (*S*=0.474), ‘Loss of family’ (*S*=0.3432), ‘Academic stressors’ (*S*=0.3399), and ‘Poverty (*S*=0.3196) were endorsed moderately by the panel for specificity (**Table-3**).

In the interviews, respondents strongly raised the importance of poverty as a structural risk factor for adolescent depression. Poverty was described as not only a limitation of finances, but as lack of opportunity and absence of other resources (e.g., public goods such as libraries; educational and vocational resources; or access to these resources). Respondents stated that such conditions create and perpetuate a sense of hopelessness which can negatively affect the emotional and cognitive well-being of adolescents. One respondent shared that poverty increased susceptibility to developing cognitive distortions about the future, and imposed restrictions on possible actions that could be adopted in response. Poverty was discussed as a powerful mediator and moderator not only of depression itself, but also as a risk factor for physical illness and loss of family and close others, which could subsequently amplify psychological vulnerability:

“In my mind, poverty is the lack of opportunity, lack of resources, and not necessarily only the lack of finances, and in the context in which I practice, a lot of adolescents don't have opportunities and don't anticipate having opportunities. That, in a way, is a closed door early in life where you have no hope from progressing from a difficult situation and you are not in control of the situation, because you lack the opportunity. So, poverty for me is a broader thing that includes the environment an adolescent finds themselves in and they can't change it, and in a way they feel stuck. For the average adolescent in LMIC, they are faced with this challenge.”

Another respondent commented on the impact of poverty on interventions for depression management:

“A lot of the ways we could help adolescents decrease depressive symptoms are highly constricted by poverty, and symptoms can be so strongly perpetuated by hopelessness, [and] lack of opportunity, that it makes intervention strategies very hard.”

While discussing social relationship issues, the role of social media emerged as an area of interest in the interviews. Some respondents commented on how social media had transformed the ecological space adolescents inhabited, in contrast to previous generations. The use of popularity metrics used in social media environments was considered detrimental to adolescents’ mental health.

“Adolescents are exposed to so much on social media where a lot is related to ‘likes,’ or other metrics associated with popularity – things like that. Some toxic group dynamics [are present in social media], so that's also a factor. And the amount of time that is spent on [social media] are contributing to worsening outcomes. Compared to traditional adolescent experiences nowadays they are exposed to so much in the world, prospects for life satisfaction, employment etc. these affect them more than before. They are almost being expected to think as adults, and think of themselves as adults, and as such there are challenges to cope.”

Respondents shared that social media may act as the trigger for risky behavior (e.g., ‘copycat suicides’). However, some respondents mentioned the value of social media to create awareness, measure social connectivity, and as a platform for preventative interventions.

Although ‘gender roles and expectations’ were not endorsed in the ranking exercise, several respondents raised the crucial role this risk factor plays in adolescent depression. Respondents discussed difficulties faced by girls in parts of the world where patriarchal norms dominate, and roles of women are marginalized. These conditions increase their risk of depression. Expectations for boys to take on financial and family responsibility at early ages were cited by respondents as creating conditions of stress and pressure, leading to the worsening of their mental health. One respondent observed that this generation of young adolescents is growing up exposed to a ‘global norm’ of women's empowerment and the ‘independent woman.’ The juxtaposition of this norm compared to traditional gender-related ideals maintained by older generations in some cultures could increase the possibility of inter-generational conflict within families and contribute to poor mental health outcomes for girls. The respondent added that these narratives of women's empowerment often exclude boys:

“[There are] places where gender roles are rigid, and patriarchal societies where men hold power, and from young ages, boys are given more decision-making power, and given access to more resources, and have their education valued, are allowed more freedom, even in social relationships. I think those type of gender roles – the demands and restrictions placed on girls can limit their functioning and hope for the future. This can contribute to depressive symptoms or even perpetuate them. Just the very structure around how girls are expected to act and the responsibilities they're supposed to fulfill can contribute to isolation and hopelessness. On the other hand, I've seen examples of lots of stress and pressure put on boys, as they are expected to achieve and be providers, and when they are growing up in a place where those resources don't exist, or they cannot go to school, it's possible that stress starts early.”

#### 3.2.4 Early signs of adolescent depression

The panel endorsed ‘Changes in mood’ (*S*=0.5632) with high salience for specificity of early depression signs. ‘Loss of interest’ (*S*=0.4553), ‘Changes in energy’ (*S*=0.4269), ‘Social isolation’ (*S*=0.4266), ‘Suicidality and self-harm’ (*S*=0.3853), and ‘Changes in sleeping patterns’ (*S*=0.3573) were moderately endorsed for specificity. Only ‘Academic decline’ (*S*=0.5983) was endorsed highly by the panel for measurement feasibility (**Table-4**).

One key observation shared across interviews was the difficulty in using cognitive and emotional signs for identification of depression. In many cultures, adolescents or parents tend to not perceive the emotional and cognitive signs as important enough to warrant seeking treatment. The issue only becomes salient when symptoms reach the behavioral level, such as academic decline, emotional reactivity, substance abuse, self-harm or suicidal behaviors:

“…we have noticed that the inactivity typically associated with depression in other countries is not commonly reported, or perhaps not even experienced. Here people are expected to continue with their tasks. The concept of ‘less activity’ is not really relatable in [our] context. Adolescents usually express distress through substance abuse and aggression, as well as other externalizing behaviors.”

#### 3.2.5 Detection strategies of adolescent depression

Detection strategies were only ranked for feasibility. ‘School based’ strategies (S=0.6469), ‘Screening tests’ (*S*=0.5365), ‘Education and awareness’ (*S*=0.5198), and ‘Health facility’ (*S*=0.5073) based strategies all received high endorsement from the panel. ‘Home based’ (*S*=0.3) strategies received moderate endorsement (**Table-5**).

Although schools were endorsed to be the ideal platform, respondents noted that detection needs to be expanded beyond simple screening and recommended a system that initiated with teacher referral, a visit to the counselor for assessment, and subsequent referral to mental health services. One respondent observed that although school is a key component for detection, the role of cultural and community factors were often important facilitators of detection:

“I work mainly among [ethnic minority groups]. Communities are hierarchically organized, and any type of intervention requires buy-in from the chief through to the head-of-household. However, once a topic is thought to be important and permission is granted, people consent and participate in high numbers. Depression and many other mental health concepts are not well described in the [local] language. Spirit possession, ancestral unhappiness, and uncompleted rituals all form part of the dominant narrative describing mental health in the community.”

## 4 DISCUSSION

Thirty-one risk factors for adolescent depression were generated. Panelists ranked three as highly specific and highly feasible to measure: family history of depression, exposure to bullying, and a negative family environment. Six were ranked as modestly specific and highly feasible: physical illness or disability, female sex, bereavement, trauma exposure, substance abuse, and low self-esteem. An additional 5 items were modestly specific and modestly feasible: social difficulties, academic stress, poverty, loss of family, and cognitive distortions. Five symptoms were at least modestly specific and feasible to measure: mood changes, loss of interest, social isolation, suicidality, and sleep changes. Schools were considered the most feasible place for screening.

The results reveal valuable insights from a global perspective on risks surrounding adolescent depression. The identification of risk factors specific to adolescent depression and the inclusion of feasibility rankings offers important criteria when considering initiatives for risk detection and prevention. For example, although the results are clear on the importance of family history for adolescent depression, the challenge of measuring family history accurately hinders its capability to function as a reliable risk factor. Other results provide context to understanding current directions in depression detection. For example, the inclusion of inflammatory markers as a biological risk factor in the ranking exercise reflects recent research which has indicated an interesting association between inflammatory markers and depression (Dantzer, O'Connor, Lawson, & Kelley, 2011; Miller & Raison, 2016). The actual panel rankings and respondent insights, however, demonstrate a critical skepticism towards this particular direction of understanding psychopathology and the need for further research in this area. When considering structural risk factors, the results indicate the crucial role of poverty and societal gender roles in shaping the environment which adolescents inhabit, and how these negatively shape the psychology of adolescents through the conditions of limitations and deprivation.

Important cultural factors included structural elements such as societal norms, family structures and hierarchies, stigma, and gender roles and expectations. Respondents discussed the importance of these factors in framing the experience of adolescence, moderating the risk of adolescent depression, and associated sick role actions. Additionally, culture was discussed as highly influential in conditioning the trivialization of certain cognitive and emotional early signs, while norming certain externalizing behaviors and coping strategies. Parents and family members are the closest stakeholders to adolescents and the home environment would theoretically be an opportune place to detect depression early. However, the findings reflect panelists’ low ranking of the home as a location to detect depression. Poor mental health literacy and stigma could potentially be the factors driving the unreliability of home-based detection with parents as unreliable raters of depressive behavior among adolescents. The earliest signs and symptoms were indicated by panelists to be subtle cognitive or affective changes, and easily conflated with normal mood fluctuations during adolescence, making these difficult to detect. Panelists also reported that adolescent-parent relationships often suffer from poor communication related to mental health issues, potentially further complicating the issue of home-based detection. The lack of consensus around the profile of psychological risk factors presented for ranking in this study could be reflective of global variations in symptomology, and how context and culture moderate which symptoms manifest, are considered of importance, and therefore, are communicated. The heterogeneity in results of psychological risk factors also highlight the challenges in current mental disorder classification systems rooted in the assumption of reified stable disease entities which persist across populations and societies (Clark, Cuthbert, Lewis-Fernandez, Narrow, & Reed, 2017). Based on these results, we present several policy and programmatic recommendations, and areas of further research, in Box 1.

**Box 1:** Policy and Programmatic Recommendations

**Recommendation 1:** Longitudinal cohort studies are necessary to understand the comprehensive and causal links between biological risk factors and the manifestation and course of adolescent depression, in multiple global settings.
**Recommendation 2:** New procedures of measuring family history are necessary. Standardized instruments should be developed, culturally validated, and implemented with both parents and secondary relatives to better identify at-risk adolescents. Idioms of distress should be elicited and utilized to make such instruments contextually sensitive.
**Recommendation 3:**Psychological risk factors remain poorly understood. Longitudinal cohort studies are recommended in multiple global settings to understand the complex pathways via which psychological factors cause or moderate adolescent depression. These need to be complemented by qualitative studies which can elucidate the role of cultural and contextual factors.
**Recommendation 4:** To better inform clinical practice, a risk calculator of adolescent depression needs to be developed to identify at-risk youth who could benefit from preventive services and to categorize youth with depression by risk profiles that could inform more tailored treatment.
**Recommendation 5:** Social media represents a new ecological space inhabited by adolescents of this and subsequent generations. This new space presents with risks and also opportunities. Research needs to be done on the potential of social media as a medium for awareness and detection, and on the connection of social media with increased risk for depression.
**Recommendation 6:** Culture and context play a vital role in risk, manifestation, prevention and treatment of adolescent depression. Accordingly, cultural adaptation for risk detection, screening, and treatment regimens are necessary. Identification of universal risk factors will require contextualization for assessment across populations and settings.
**Recommendation 7:** School is an ideal platform for detection due to the presence of stakeholders like teachers and counselors who can facilitate risk detection, screening, referral and treatment for adolescent depression. These need to be complemented by efforts to reach adolescents not enrolled in school.

### 4.1 Strengths and limitations

This Delphi study has several strengths. First, a major strength is the inclusion of experts from multiple countries across the world. This makes the results compelling as it provides a set of endorsed items which constitute a global perspective of depression risk. Considering the large gaps in the literature on adolescent depression in LMIC, this makes the results of this study a valuable contribution. The inclusion of variables from the preliminary results of a systematic review adds empirical strength to the ranking exercise. The use of the Smith's Salience index provides a more robust indicator of consensus, than the use of simpler centrality metrics that are commonly reported in Delphi studies. The use of qualitative interviews to gain detailed insight into the response patterns of the panel is a major strength, allowing enhanced understanding of the statistical results, and nuanced commentary on the important influence of culture. The study has limitations in that the panel was comprised of primarily psychiatrists or psychologists, and did not include other professions like social workers, counselors, and pediatricians. Secondly, despite extensive efforts, participants were not representative of all countries and cultural regions. Another limitation is that risk factors were grouped together in biological, psychological, and environmental domains. Some risk factors could have been fit within multiple groups, and the particular categorization may have led to different interpretations of the items.

## 5 CONCLUSION

This Delphi study provides recommendations for biopsychosocial risk factors, early signs, and detection strategies for adolescent depression from a global perspective. The expert consensus established in this study can inform areas of future research in adolescent depression. By generating consensus on both specificity and feasibility for adolescent depression, this study provides an actionable set of indicators for inclusion in depression detection, assessment and risk prevention initiatives. The results from the ranking exercise were supplemented with detailed commentary via qualitative interviews which provides valuable cultural considerations on challenges and solutions identified in the results. Clinically, these indicators can inform future work on the development of risk calculators for early identification efforts and to characterize risk profiles that could shape different approaches to prevention (Kieling et al., 2019). The set of risk factors identified in this study can inform research and clinical practice across diverse global settings to generate an evidence base that can inform both local and universal perspectives of risk and prevention of depression among the world's 1.2 billion adolescents.

## Author contributions

BAK, HLF, CK, and VM conceived the paper and were responsible for overall direction and planning. SSW, KO, RH, and BAK wrote the manuscript with input from all the authors. SSW developed the Delphi tool and conducted the quantitative analyses. SSW, KO, and RH conducted the qualitative analyses. KG, HLF, CK, and VM provided overall technical review, critical revision, and final approval for publication.

## Study Dates

The dates of the IDEA Delphi study were from March 2019 to February 2020.

## Declaration of Competing Interest

We declare that we have no competing interests.

## Appendix Supplementary materials

Image, application 1
